# The ProtekDuo Cannula: A Comprehensive Review of Efficacy and Clinical Applications in Right Ventricular Failure

**DOI:** 10.3390/jcm13144077

**Published:** 2024-07-12

**Authors:** Joseph M. Brewer, Marc O. Maybauer

**Affiliations:** 1 Specialty Critical Care and Acute Circulatory Support Service, Nazih Zuhdi Transplant Institute, INTEGRIS Health Baptist Medical Center, Oklahoma City, OK 73112, USA; 2 Division of Critical Care Medicine, Department of Anesthesiology, University of Florida, Gainesville, FL 32610, USA; 3 Department of Anaesthesiology and Intensive Care Medicine, Philipps University, 35037 Marburg, Germany; 4 Critical Care Research Group, Prince Charles Hospital, University of Queensland, Brisbane, QLD 4072, Australia

**Keywords:** ProtekDuo, right ventricular failure, right ventricular assist device, RVAD, mechanical circulatory support

## Abstract

Right ventricular failure (RVF) is a clinical challenge associated with various underlying acute and chronic medical conditions, necessitating diverse management strategies including mechanical circulatory support (MCS). The ProtekDuo cannula represents an important advancement in medical devices for MCS in the setting of RVF. When combined with an extracorporeal blood pump, the dual-lumen design allows for direct bypass of the RV using simultaneous drainage and return of blood using percutaneous, single-site access. Studies have reported favorable outcomes with the ProtekDuo cannula and low device-related complications, but comparative studies with other MCS devices are limited. Still, the ProtekDuo cannula has numerous advantages; however, it is not without challenges, and opportunities for further research exist. The ProtekDuo cannula holds significant potential for future advancements in the field of MCS, offering promising solutions for RVF management.

## 1. Introduction

Right ventricular failure (RVF) is a complex clinical challenge [1,2], associated with numerous conditions including acute myocardial infarction (MI), pulmonary embolism (PE), post-cardiotomy including after left ventricular assist device (LVAD) and heart transplant, acute respiratory distress syndrome (ARDS), and others [3,4,5,6,7,8,9,10,11]. The diverse etiologies and significant impact on patient morbidity, mortality, and healthcare costs underscore the need for timely and varied management strategies for RVF [2,8,11,12,13,14,15,16,17,18,19].

Medical management utilizing inotropes to improve myocardial contractility, diuretics to optimize volume and preload, and pulmonary vasodilators to control afterload remains the mainstay for the treatment of RVF [12,18,20,21,22]. However, when RVF is refractory to medical management, mechanical circulatory support (MCS) is often employed [22,23]. For many patients with acute RVF supported with MCS, recovery of function, device explantation, and survival is possible; however, these factors depend heavily on the device used, the timing of support initiation relative to the development of RVF, and the severity of illness of the recipient [12,20,22,24,25,26,27,28].

The use of venoarterial (VA) extracorporeal membrane oxygenation (ECMO), which provides biventricular support, has been utilized in cases of RVF [25,29,30]. However, VA ECMO may lead to maladaptive and detrimental cardiorespiratory effects. Additionally, complications have been more frequent, and outcomes have been poorer in certain patients with RVF when compared to those receiving RVAD support [25,29]. Consequently, in scenarios of isolated RVF or biventricular failure with left ventricle (LV) MCS in situ, the strategy of univentricular support has gained adoption [22,31,32].

Targeted RV MCS can be placed either centrally via surgical techniques or peripherally by percutaneous methods [21,22,29,33,34,35,36]. Surgical RVAD (sRVAD), however, is highly invasive [18,28,33] and may have a higher incidence of bleeding and infection, especially if sternal closure is delayed [33]. Additionally, the time until placement of an sRVAD can be delayed compared to the placement of a percutaneous RVAD [37].

Recent advancements have introduced specialized devices and cannulas that facilitate RV support through a single percutaneous access site, significantly reducing the invasiveness required to manage refractory RVF [18,28,38,39,40,41,42]. One such device is the ProtekDuo (LivaNova, London, UK) cannula. When used in combination with an extracorporeal blood pump, it serves as a temporary percutaneous RVAD (tpRVAD), offering a promising solution for RVF challenges [4,43].

As the utilization of the ProtekDuo cannula increases, recognizing the evidence supporting its use becomes increasingly important [22,43,44,45,46]. This review aims to describe the clinical applications, efficacy, and safety of the ProtekDuo cannula in managing RVF. It also compares outcomes with other common tpRVADs when possible, highlights its important role, and outlines directions for future research.

## 2. Literature Review

Currently, data on most clinical outcomes of interest regarding the ProtekDuo cannula as a tpRVAD are primarily derived from case reports, case series, and retrospective cohort studies [43,44,45]. This section reviews much of the existing research on the ProtekDuo cannula as a tpRVAD, focusing on retrospective cohort studies, which are currently the highest level of available evidence, and key outcomes such as hemodynamic stability, complications, and survival rates. It begins with studies comparing the ProtekDuo cannula to other temporary percutaneous cannulation configurations or devices (group 1). The review then discusses studies involving patients supported exclusively by the ProtekDuo cannula, without comparison to other tpRVADs (group 2), from which valuable insights can also be derived.

### 2.1. Group 1: Comparative ProtekDuo Studies

The retrospective cohort studies comparing the outcomes of the ProtekDuo cannula with other tpRVAD devices and configurations are limited to single-center settings, and the participant sizes range from 24 to 58 patients. All studies involve patients with RVF stemming from various etiologies.

Of the three studies comparing patients supported with ProtekDuo cannula versus the Impella RP in patients with multiple etiologies of RVF, only one reported better survival in the ProtekDuo-supported group [26] and two reported no difference in survival between the two device groups [22,47]. Meanwhile, the single study comparing patients supported with a ProtekDuo cannula to patients supported with a two-cannula configuration tpRVAD also reported no significant difference in survival [3]. Two studies reported improved hemodynamics for all patients; however, changes in hemodynamic parameters were not compared by device type [22,26] (Table 1).

Lastly, two studies reported and compared device-related complications by device type [3,47]. Ritter et al. reported significantly fewer bleeding and ischemic complications in the ProtekDuo-supported cohort compared to the two-cannula-supported cohort [3], whereas Agrawal et al. found no significant difference in complications between patients supported with a ProtekDuo cannula compared to those supported with an Impella RP [47]. George et al. reported complications but did not specify whether they were device-related or non-device-related, nor did they indicate which device was associated with these complications [22] (Table 1).

### 2.2. Group 2: Non-Comparative ProtekDuo Studies

Numerous retrospective cohort studies have evaluated the ProtekDuo cannula as a tpRVAD without comparison to other tpRVADs. Like those comparing outcomes between devices, the majority of these studies were conducted at a single center, with sample sizes ranging from 10 to 40 patients [4,5,6,8,9,10]. One exception was a study that utilized a dataset from a large cardiogenic shock registry for the analysis of 159 patients [48]. Two studies focused exclusively on patients with post-LVAD RVF [9,10], while another only included patients with MI [8]. The remaining studies involved patients with RVF due to a variety of causes [4,5,6,48] (Table 1).

All studies of the ProtekDuo cannula as a tpRVAD reported survival rates at various time points. Four studies noted 30-day survival rates between 60% and 85.2% [6,8,9,10], three reported 1-year survival rates from 60% to 81.5% [8,9,10], and three indicated survival-to-discharge rates from 60% to 85.2% [4,8,9]. In the study by Badu et al., survival-to-discharge rates varied significantly based on the etiology of RVF: 88.9% for post-cardiotomy patients, 41.7% for MI or heart failure patients, and 60% for patients with respiratory failure [4]. Hernandez Montfort et al. reported an in-hospital survival rate of 48% [48]. Two studies documented survival-to-weaning rates between 72.5% and 88.9% [4,9], and three studies reported survival at either 60, 90, or 180 days [5,6,10] (Table 1).

Four studies reported significant improvement in hemodynamic parameters by a reduction in dose or overall number of vasopressors and/or inotropic medications [4,9], CVP [4,5,8,9], increase in mean arterial pressure [5], and an increase in central venous oxygen saturation [8]. Lim et al., however, reported no significant change in vasopressor or inotropic dose in their group of patients [5]. Three studies did not report changes in hemodynamic parameters [6,10,48] (Table 1).

Five studies reported either device-related or non-device-related complications in patients supported with a ProtekDuo cannula. Two studies found no device-related complications [8,10], whereas others reported complications including cannula migration [4,9], superior vena cava (SVC) syndrome [4], right internal jugular vein (RIJV) thrombus [4,9], and moderate to severe tricuspid regurgitation [9]. Four studies reported non-device-related complications including acute kidney injury (AKI) requiring renal replacement therapy (RRT) [6,8], postoperative bleeding [8], gastrointestinal bleeding [6], stroke [6,10], hemolysis [9], sepsis [6,8], and conversion to sRVAD [9]. AKI requiring RRT was the most commonly reported non-device-related complication (Table 1).

### 2.3. Literature Synthesis

Significant variability exists regarding survival outcomes of patients supported with a ProtekDuo in group 1 compared to group 2 studies. When comparing similar time points including survival to weaning, hospital discharge, 30 days, and 1 year, group 1 studies reported lower survival compared to group 2 studies [3,4,6,8,9,10,22,47].

In studies reporting hemodynamic changes, the use of a tpRVAD resulted in improvement in hemodynamic parameters both in terms of right-sided filling pressure and vasopressor and/or inotropic medication requirements. These study findings were consistent in both group 1 and group 2 studies.

In terms of complications, similar complications were reported between group 1 and 2 studies including device malposition and cannula migration, both intravascular and intra-cannula thrombotic complications, and the development of moderate to severe tricuspid regurgitation [3,4,9,47]. Cannula migration and malposition were reported in similar proportions in group 1 and 2 studies. Thrombotic complications in patients with a ProtekDuo cannula were reported at higher rates in group 1 studies [3,4,9,47], while the development of tricuspid regurgitation was reported at a higher rate in group 2 studies [9,47]. Superior vena cava syndrome was not reported in group 1 studies [4], whereas bleeding and infection were not reported in group 2 studies. Both group 1 and 2 studies reported AKI requiring RRT in similar proportions and stroke, which occurred in higher proportions in group 2 studies [6,8,10,22].

## 3. Discussion

The available research on the ProtekDuo cannula is predominantly limited to retrospective cohort studies, which often include a small number of participants. These studies represent the highest level of evidence for assessing the ProtekDuo cannula both independently and in comparison to other tpRVAD devices or configurations. Survival rates at various time points were frequently lower in group 1 studies. However, improvements in hemodynamics and vasopressor and/or inotrope requirements were consistently observed in both group 1 and 2 studies. Both groups of studies reported the occurrence of device- and non-device-related complications at similar rates, including device malposition and cannula migration, thrombotic complications, and the development of tricuspid regurgitation.

Only one study utilized multicenter data from a large registry, offering the benefit of substantial data volume but potentially compromised by the absence of randomization and variability in collection, completeness, and quality. The remaining single-center, retrospective cohort studies exhibit inherent limitations, including variations in patient demographics and clinical practices such as MCS indications, contraindications, and weaning criteria. These factors may limit the generalizability of the findings, introduce potential selection bias, and reduce control over variables due to the lack of a control group or randomization. Furthermore, researcher bias may also affect the objectivity of the data analysis in these retrospective studies. In sum, these limitations affect the overall strength of conclusions that can be made regarding the comparative efficacy of the ProtekDuo cannula compared to other tpRVAD devices or cannulation configurations.

### 3.1. Clinical Use of the ProtekDuo Cannula

**Figure 1:**
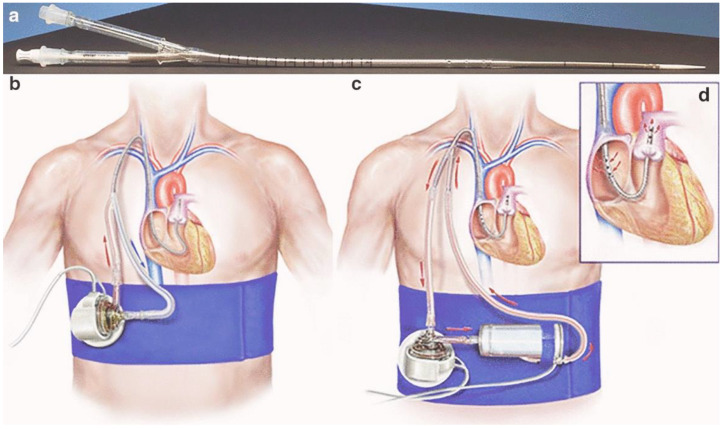
ProtekDuo dual-lumen cannula. (**a**) ProtekDuo cannula with introducer; (**b**) ProtekDuo cannula connected to extracorporeal blood pump; (**c**) ProtekDuo cannula connected to extracorporeal blood pump with interposed membrane lung; (**d**) ProtekDuo cannula demonstrating blood drainage in the right atrium from the proximal outer cannula and blood return into the main pulmonary artery from the distal inner cannula. From Condello 2020 [50]. Creative Commons license http://creativecommons.org/licenses/by/4.0/.

The dual-lumen ProtekDuo cannula, known for its innovative design and potential efficacy in treating RVF [4,34,49], is typically placed in the RIJV. The cannula is available in two sizes: 31 French (Fr) and 29 Fr. The unique cannula-within-a-cannula design enables simultaneous, omnidirectional drainage and return of blood through two distinct lumens (Figure 1a–d). The outermost proximal cannula, measuring 28 cm, features 16 circumferential side holes for drainage. The innermost distal cannula, extending the entire length of the cannula, allows for blood return through an end hole and six side holes at the terminal portion (Figure 1a,d). The design allows for the direct bypass of the RV, delivering 4–4.5 L (L) of flow for the 29 Fr, and 4.5–5 L for the 31 Fr cannula, from the right atrium (RA) to the main pulmonary artery (PA) (Figure 1d).

#### 3.1.1. ProtekDuo Insertion

The ProtekDuo cannula is placed using the modified Seldinger technique. After placing an 8 Fr to 9 Fr introducer sheath in the RIJV, a 7 Fr balloon-tipped catheter is inserted through the sheath and directed into the right PA. Next, a 0.035-inch/260 cm Lunderquist extra stiff guidewire (Cook Medical, Bloomington, IN, USA) is introduced through the balloon-tipped catheter and advanced into the right PA under fluoroscopic guidance. The balloon-tipped catheter and introducer sheath are carefully withdrawn over the wire using fluoroscopy to ensure the wire remains positioned in the right PA. If indicated, a bolus of unfractionated heparin (UFH) is administered to achieve the desired activated clotting time or partial thromboplastin time. The insertion site is progressively dilated, and the cannula is inserted under continuous fluoroscopic guidance until the tip of the cannula is in the proximal portion of the right PA. The wire and inner cannula dilator are carefully removed, and the cannula lumens are connected to the extracorporeal circuit. Pump flow is gradually increased and the positioning of the cannula tip within the main PA is confirmed. The presence of the cannula tip within the main PA can be further confirmed using transesophageal echocardiography (TEE). Once the final cannula position is assured, the device can be sutured securely to the patient [51].

#### 3.1.2. ProtekDuo Weaning and Removal

When a ProtekDuo cannula is used as a tpRVAD, weaning should occur promptly once the patient’s clinical condition stabilizes, with improved RV function by echocardiography, hemodynamic, and organ function parameters, and reduced requirements for vasopressor and inotrope support [52,53]. There is no standardized approach to tpRVAD weaning, regardless of the device used; however, one method involves gradually reducing pump flows in small increments (e.g., 0.25 to 0.5 L/min) until a stable flow rate of approximately 2 L/min (or minimum speed of 3500 rpm) is tolerated [52,53]. At that time, a bedside “turn down” test is conducted by further decreasing flows in 0.5 L/min increments until complete flow cessation is reached, typically by clamping the return limb of the circuit. Prior to reducing flows below 2 L/min, a bolus of UFH is given to maintain an activated clotting time (ACT) of 250–300 s and prevent clot formation during the low-flow state. Baseline echocardiographic and hemodynamic parameters are compared to repeated parameters following each step of the turn-down. If significant RV dysfunction or hemodynamic instability is observed, tpRVAD support should be continued. If the patient remains clinically stable, the cannula can be safely removed at the bedside [8,9,10,51,52,53].

### 3.2. ProtekDuo Cannula Advantages

The ProtekDuo cannula offers several advantages for managing RVF patients requiring MCS [43]. First, the single-cannula design facilitates percutaneous implantation, eliminating the need for complex surgeries or cardiopulmonary bypass procedures, which may promote more timely initiation of RVAD support [8,9,10]. Additionally, the single-site access in the upper body (Figure 1b,c) eliminates the need for devices or cannulas in the groin vessels, thus allowing for improved patient mobility [4,8,9,10,12,23,28,54,55,56], which is associated with better outcomes [57,58,59] (Table 2).

A ProtekDuo-based tpRVAD configuration allows for the interposition of a membrane lung (ML) (Figure 1c), which is not possible in tpRVAD systems that utilize an intracorporeal axial flow pump (Table 2) [44]. However, concomitant respiratory support via an ML may be required in 10–50% of patients with RVF [4,6,8,9,10,19,28,48,56]. Additionally, the ability to provide comprehensive respiratory support may allow for earlier extubation or improved tolerance of lung protective ventilation in patients with concurrent respiratory failure [21].

Finally, the use of the ProtekDuo cannula for RVF may allow for the avoidance of peripheral venoarterial (VA) ECMO and its potential complications including lower extremity ischemia, LV distension, aortic root thrombus, and upper body hypoxia [28,29,60,61]. Additionally, physiological anterograde pulsatile flow from the left ventricle is maintained [8]. These combined attributes make the ProtekDuo cannula a promising tool for RVF management, addressing clinical challenges while optimizing patient outcomes.

### 3.3. ProtekDuo Cannula Challenges

Despite its advantages, the ProtekDuo cannula faces challenges from both extrinsic and intrinsic (device-related) factors. Primarily, when used as a tpRVAD, the cannula provides support solely for the RV and is thus suitable only for cases of isolated RVF. In scenarios where LV failure coexists, the increased LV preload facilitated by the ProtekDuo can lead to LV volume overload, pulmonary edema, and respiratory failure [21]. Consequently, in the presence of significant LV dysfunction, an MCS strategy that provides biventricular support is most appropriate [11,25,30,62].

Additionally, successful implantation of the ProtekDuo cannula depends on specific anatomical requirements [21]. The patient must have an accessible and patent RIJV, although access using alternative sites such as the left subclavian vein has been reported [63]. The pathway from the accessed vessel to the main PA must be free from obstruction, such as venous stenoses, thrombi, and valvular abnormalities [21].

Anatomical or physiological factors can also limit the cannula’s performance. Effective operation requires competent valves to separate blood drainage and return portions of the cannula [21]. Pulmonic insufficiency may lead to refractory RV distention and failure as well as persistent hypoxia resulting from recirculation due to reduced ML efficiency. Severe pulmonary hypertension could impede pump performance and diminish forward blood flow through the pulmonary circulation, potentially causing RV volume overload and LV underfilling [21]. Lastly, the negative pressure created by drainage in the RA could induce left-to-right shunting if a large patent foramen ovale or atrial septal defect is present [21]. In such cases, VA ECMO may offer more suitable support.

Common complications associated with large cannulas including device migration, thrombosis, vascular injury, and cannulation site bleeding have been reported [4,7,9,47]. Additionally, uncommon complications have been reported including SVC syndrome [64], fracture of the cannula during extended use [65], right coronary artery compression [66], and intracannula thrombus formation [67], emphasizing the need for comprehensive safety and long-term performance studies.

Implantation of the ProtekDuo cannula, like other dual-lumen cannulas, is considered more complex than implantation of single-lumen cannulas [68,69]. Percutaneous implantation, although beneficial, necessitates skilled teams and advanced imaging technology, posing challenges for some centers. Patient selection criteria should be carefully assessed for optimal outcomes. Moreover, cost considerations and resource availability may hinder widespread adoption.

Finally, the lack of randomized controlled trials (RCTs) is a notable challenge to the selection and application of the ProtekDuo cannula (and other tpRVAD devices and cannulation configurations) for patients with RV failure requiring MCS. 

## 4. Future Directions

The ProtekDuo cannula, with its unique design, promising advantages, and increasing clinical experience, holds significant potential for future advancements and innovations in the field of MCS. The use of the ProtekDuo cannula has expanded significantly over time, extending beyond RVAD applications. Maybauer et al. described numerous additional configurations and applications possible with the cannula [34]. These include ECMO for respiratory failure [70,71,72,73,74], venous drainage for cardiopulmonary bypass [75], VA ECMO [76,77], and its use in conjunction with other MCS devices for biventricular support [62,75,78,79,80,81,82]. Some authors have even described its use as an LVAD via transapical placement [83,84,85] and as a biventricular assist device [86].

Future research should address the notable gap in the current literature by prioritizing large-scale RCTs aimed at assessing patient outcomes, quality of life, and long-term survival with the ProtekDuo cannula, especially compared to other tpRVAD devices and configurations. Additionally, future research should focus on refining patient selection criteria based on specific clinical profiles and hemodynamic parameters. Tailored approaches for different populations, including pediatric and adult populations, may enhance the effectiveness of the cannula. Lastly, research should focus on incorporating the ProtekDuo cannula into long-term support strategies while enhancing the device’s long-term durability. Related to this area are studies focused on minimizing device-related complications and adverse events with the potential of using innovative techniques and materials to contribute to safer and more reliable device performance.

## 5. Conclusions

The clinical management of RVF is complex, and multiple MCS options including tpRVADs are available as support tools when medical management alone is insufficient. The ProtekDuo cannula has shown promise as an intervention for RVF, with multiple studies demonstrating its clinical efficacy and safety in various contexts; however, knowledge gaps still exist. Despite challenges and limitations, the ProtekDuo cannula offers important advantages for a tpRVAD such as non-surgical implantation, improved patient mobility, compatibility with MLs, and diverse applications. Additional high-quality research is needed that is focused on refining patient selection criteria and assessing long-term outcomes of tpRVAD support. Finally, research that evaluates the potential applications of the ProtekDuo cannula beyond tpRVAD support is needed.

## Figures and Tables

**Table 1:** Key outcomes of included studies.

First Author, Year	RVF Etiologies	Sample Size	Hemodynamic Changes	Complications n (%)	Survival n (%)
Group 1: Comparative ProtekDuo Studies					
Agrawal, 2021 [47]	PCS, post-LVAD, MI, PE, PGD	28 ProtekDuo: 14 Impella RP: 14	NR	*DRC:* Malposition: ProtekDuo: 1 (7.1) Impella RP: 2 (14.3) Thrombosis: ProtekDuo: 2 (14.3) Impella RP: 2 (14.3) Hemolysis: ProtekDuo: 0 (0) Impella RP: 2 (14.3) Severe TR: ProtekDuo: 0 (0) Impella RP: 1 (7.1)	Weaning: ProtekDuo: 9 (64.3) Impella RP: 9 (64.3) Hospital discharge: ProtekDuo: 5 (35.7) Impella RP: 5 (35.7) 1-year: ProtekDuo: 4 (28.6) Impella RP: 4 (28.6)
Ritter, 2023 [3]	Post-LVAD, PCS, HF	24 Two-cannulas: 12 ProtekDuo: 12	NR	*DRC:* Bleeding: * All patients: 14 (58.3) Two-cannula: 10 (83.3) ProtekDuo: 4 (33.3) Thrombosis: All patients: 6 (25.0) Two-cannula: 4 (33.3) ProtekDuo: 2 (16.7) Ischemia: * All patients: 5 (20.8) Two-cannula: 5 (41.7) ProtekDuo: 0 (0) Infection: All patients: 16 (66.7) Two-cannula: 8 (66.7) ProtekDuo: 8 (66.7) Neurological complication: All patients: 3 (12.5) Two-cannula: 3 (25.0) ProtekDuo: 0 (0)	ICU discharge: All patients: 13 (54.2) Two-cannula: 6 (50.0) ProtekDuo: 7 (58.3) Hospital discharge: All patients: 12 (50.0) Two-cannula: 5 (41.7) ProtekDuo: 7 (58.3)
George, 2023 [22]	PCS, post-LVAD, MI, COVID-19 respiratory failure, HF, PE, other	42 ProtekDuo: 32 Impella RP: 6 sRVAD: 4	All patients: Significant reduction in median number vasopressor or inotropes throughout support	Major bleeding: All patients: 23 (54.8) Stroke: All patients: 3 (7.1) AKI requiring RRT: All patients: 18 (42.9)	90 days: All patients: 16 (38.1) Impella RP: 2 (33.3) ProtekDuo: 11 (34.4) sRVAD: 3 (75.0) 1-year: All patients: 10 (23.8) Impella RP: 1 (16.7) ProtekDuo: 7 (21.9) sRVAD: 2 (50.0)
Gupta, 2024 [26]	HF, complex PCI, ACS, PE, sepsis	58 ProtekDuo: 29 Impella RP: 29	All patients: Significant reduction in CVP and increase in CI at 24 h	NR	In-hospital: * Impella RP: 10 (34.5) ProtekDuo: 20 (69.0)
Group 2: Non-Comparative ProtekDuo Studies					
Schmack, 2019 [10]	Post-LVAD	11	NR	*DRC:* None *Non-DRC:* Hemorrhagic stroke: 1 (9.0)	30 d: 8 (72.7) 60 d: 7 (63.6) 360 d: 7 (63.6)
Badu, 2020 [4]	PCS (including post-LVAD), MI, HF, hypoxemic respiratory failure	40 PCS: 18 MI- or HF-CS: 12 Hypoxemic respiratory failure: 10	Significant reduction in both VIS and CVP	*DRC:* Cannula migration: 3 (7.5) SVC syndrome: 3 (7.5) RIJV thrombus: 1 (2.5) *Non-DRC:* NR	Weaning: All patients: 29 (72.5) Postcardiotomy: 17 (94.4) Cardiogenic shock: 5 (41.7) Respiratory failure: 7 (70.0) Discharge: All patients: 27 (67.5) Postcardiotomy: 16 (88.9) Cardiogenic shock: 5 (41.7) Respiratory failure: 6 (60.0)
Kremer, 2020 [8]	MI	10	Significant reduction in CVP and increase in ScvO2	*DRC:* None *Non-DRC:* AKI requiring RRT: 8 (80.0) Post-operative bleeding: 4 (40.0) Hemorrhagic stroke: 1 (10.0) Organ ischemia: 1 (10.0) Infection/sepsis: 4 (40.0)	Discharge: 6 (60.0) 30 d: 6 (60.0) 1-year: 6 (60.0)
Lim, 2020 [5]	Post-LVAD, post-heart transplant, HF	11	Significant reduction in CVP and increase in MAP ^a^ No significant change in vasopressor or inotropic dose ^a^	*DRC:* NR *Non-DRC:* NR	90 d: 7 (63.6)
Salna, 2020 [9]	Post-LVAD	27	Significant reduction in CVP at 6 h, 12 h, and 48 h after ProtekDuo insertion Significant reduction in number of vasopressors at 6 h, 12 h, and 48 h after ProtekDuo insertion Significant reduction in epinephrine, norepinephrine, and vasopressin doses at 6 h after ProtekDuo initiation Significant reduction in milrinone dose at 48 h after ProtekDuo initiation	*DRC:* Mod-severe TR: 8 (36.4) ^b^ Cannula migration: 2 (7.4) Device thrombosis: 1 (3.7) *Non-DRC:* Hemolysis: 4 (14.8) Conversion to surgical RVAD: 3 (11.1)	Weaning: 24 (88.9) Discharge: 23 (85.2) 30 d: 23 (85.2) 1-year: 22 (81.5)
Oliveros, 2021 [6]	Post-LVAD, PCS, MI, PE, post-partum CM, ARDS, post-lung resection	11	NR	*DRC:* NR *Non-DRC:* AKI requiring RRT: 5 (45.4) GI bleeding: 5 (45.4) HIT: 6 (54.5) Stroke: 2 (18.2) Sepsis: 7 (63.6)	30 d: 9 (81.8) 180 d: 7 (63.6)
Hernandez Montfort, 2024 [48]	MI, HF	159	NR	NR	In-hospital: 77 (48)

Abbreviations: ACS = acute coronary syndrome, AKI = acute kidney injury, ARDS = acute respiratory distress syndrome, CI = cardiac index, CM = cardiomyopathy, CS = cardiogenic shock, CVP = central venous pressure, d = days, DRC = device-related complications, GI = gastrointestinal, h = hour(s), HF = heart failure, HIT = heparin-induced thrombocytopenia, ICU = intensive care unit, LVAD = left ventricular assist device, MAP = mean arterial pressure, MI = myocardial infarction, NR = not reported, PCI = percutaneous coronary intervention, PCS = post-cardiotomy shock, PE = pulmonary embolism, PGD = primary graft dysfunction, RIJV = right interval jugular vein, RRT = renal replacement therapy, RVAD = right ventricular assist device, ScvO2 = central venous oxygen saturation, sRVAD = surgical RVAD, SVC = superior vena cava, TR = tricuspid regurgitation, VIS = vasopressor-inotrope score. ^a^ Seven patients in final analysis. ^b^ Echocardiogram available for 22 patients. * *p* < 0.05.

**Table 2:** Comparison of key features of common temporary RVADs.

Device	Invasiveness	Placement Complexity	Placement Requirements	Single Site	Flow L/min	Oxygenator	Ambulation
Surgical RVAD	Highly	High	Sternotomy or thoracotomy	No	>5	Yes	Yes
Impella RP	Minimally	Moderate	Real-time imaging to guide placement	Yes CFV	<4	No	No
ProtekDuo	Minimally	Moderate	Real-time imaging to guide placement	Yes RIJV	<4.5	Yes	Yes
Spectrum Cannula	Minimally	Moderate	Real-time imaging to guide placement	Yes RIJV	<4	Yes	Yes
Two-cannula configuration	Minimally	Low	Ultrasound for vascular access	No	<5	Yes	Yes

Abbreviations: CFV = common femoral vein, L = left, RIJV = right internal jugular vein, RVAD = right ventricular assist device.
